# Personalizing physical exercise in a computational model of fuel homeostasis

**DOI:** 10.1371/journal.pcbi.1006073

**Published:** 2018-04-26

**Authors:** Maria Concetta Palumbo, Micaela Morettini, Paolo Tieri, Fasma Diele, Massimo Sacchetti, Filippo Castiglione

**Affiliations:** 1 Institute for Applied Computing (IAC) “Mauro Picone”, National Research Council of Italy, Rome, Italy; 2 Department of Information Engineering, Università Politecnica delle Marche, Ancona, Italy; 3 Department of Movement, Human and Health Sciences, University of Rome “Foro Italico”, Rome, Italy; University of California Irvine, UNITED STATES

## Abstract

The beneficial effects of physical activity for the prevention and management of several chronic diseases are widely recognized. Mathematical modeling of the effects of physical exercise in body metabolism and in particular its influence on the control of glucose homeostasis is of primary importance in the development of eHealth monitoring devices for a personalized medicine. Nonetheless, to date only a few mathematical models have been aiming at this specific purpose. We have developed a whole-body computational model of the effects on metabolic homeostasis of a bout of physical exercise. Built upon an existing model, it allows to detail better both subjects’ characteristics and physical exercise, thus determining to a greater extent the dynamics of the hormones and the metabolites considered.

## Author summary

Exercise has a great impact on human metabolism and the lack of physical activity represents one of the main causes of the metabolic disorders. The effectiveness of regular physical activity in the prevention and management of several chronic diseases is widely recognized. In the study of the metabolism and related disorders, mathematical models have proven useful in describing and quantifying physiological processes often not easily measurable in vivo. Formulating a model describing the metabolic responses to a physical exercise session is a challenging task since the effects vary depending on its intensity, duration, modality and are also dependent on the subjects’ physical characteristics (e.g. age, gender, body weight, fitness status). To date, none of the existing computational models is able to provide this level of “personalization”. Thus, starting from an existing model of fuel homeostasis during exercise, we have formulated a novel computational system that is more detailed in describing both the physical exercise and the subjects’ characteristics.

## Introduction

Current mathematical models of metabolic processes can include different levels of description, details and complexity, spanning from larger, mostly qualitative to smaller quantitative models. The mathematical description of the effects of physical exercise on metabolic pathways is still a challenging task, since such effects impinge upon different key variables related to the regulation of fuel homeostasis in several organs and tissues. Moreover, the effects of exercise on the regulation of substrate metabolism are very much dependent on its intensity, duration and modality [1].

During exercise, a shift from nutrient uptake and disposal toward mobilization of stored fuels and utilization of carbohydrate and free fatty acids (FFA) entails the simultaneous regulation of insulin and the counter-regulatory hormones (i.e., glucagon, epinephrine, cortisol and growth hormone), which help matching the increased energy demand by the working muscles with substrate availability. Indeed, during submaximal exercise these hormonal responses facilitate fatty acids mobilization and oxidation, while stimulating hepatic glucose production to counteract the increased peripheral glucose uptake and to prevent hypoglycaemia [2].

To date, few mathematical models of exercise have been proposed. Some of them, e.g., the models from Breton et al., Derouich et al. and Dalla Man et al. [3–5], describe the metabolic phenomena looking at the dynamics of plasma glucose and insulin. More in details, Derouich et al. [4] combined a model for the glucose kinetics [6, 7], with a perturbed model in which three parameters represent the effect of the physical exercise in accelerating the utilization of glucose and increasing insulin sensitivity by muscles and liver. The effectiveness of this model has later been confirmed and expanded by the results of Breton [3], who proposed a “parsimonious” model of physical exercise. The model proposed by Breton relates the modifications in insulin action and glucose response to heart rate changes. In a subsequent work, Dalla Man et al. [5] proposed and tested three possible extensions of the Breton study [3]. Adamu and coworkers in [8] improved the model by Topp et al. [9] incorporating diet and physical activity as factors affecting plasma glucose and insulin kinetics. In their simulation study, exercise is modeled as the amount of calories burnt. Recently, Singh and Kumar [10] proposed a model to simulate the effect of a session of exercise on the glucose-insulin regulatory system for both non-diabetic and type 2 diabetic patients, by introducing a parameter describing glycogen breakdown and utilization during exercise. Svitra and colleagues in [11] modeled the dynamics of insulin and plasma glucose with two differential equations with one delay in a “predator-prey” fashion for normal and diabetic subjects.

Roy and Parker in [12] modeled the dynamics of glucose and insulin during short- and long-term exercise. Its advantage relies on providing a quantitative description of the physical activity in terms of relative exercise intensity expressed as percentage of maximal oxygen uptake (%VO_2max_). Kim and colleagues proposed a whole-body, multi-scale computational model [13] (hereinafter, the Kim-Seidel-Cabrera model or KSC model), incorporating cellular metabolism of different tissues/organs, to predict the responses of glucose, hormones (i.e., glucagon, insulin, epinephrine) and various substrates to moderate intensity exercise. Exercise is there described using the work rate (WR) expressed in Watt. This multi-scale model combines a set of metabolic reactions at the cellular level with a description of fuel homeostasis at the whole-body level. Its advantage leans on providing predictions on metabolite concentrations and flux rates in several tissues, something that is generally difficult to measure directly. The description of physical exercise is limited to just one exercise modality (i.e., cycling) executed at a fixed intensity (WR fixed to 125W and 60%VO_2max_ for 60 min), and performed by only one category of subjects (i.e., untrained male of 70kg).

The present work improves the KSC model by providing a better description of the physical exercise as provided by Roy et al. [12] and Kildegaard and colleagues [14] with the aim of achieving greater generalization and user-customization. In particular, we focus on the improvement of the following two aspects: i) the definition of “relative” (rather than absolute thus fixed) *exercise intensity* as well as the estimation of functional capacity in relation to age, gender, anthropometric characteristics and current fitness status; ii) modeling *oxygen consumption* and the *dynamics of epinephrine* as directly dependent on the relative exercise intensity to modulate hormones and glucose responses to different exercise modalities (e.g., cycling, walking, running, stepping).

## Models

The original formulation of the multi-scale computational model proposed in [13] describes fuel homeostasis during moderate intensity exercise below the lactate threshold (LT) by using the hormonal control to regulate cellular metabolic processes. LT refers to the intensity of exercise at which there is an abrupt increase in blood lactate levels [15]. At rest and under steady-state low-intensity exercise conditions, there is a balance between blood lactate production and utilization [16]. Above LT there is a mismatch between lactate production and uptake, with the rate of lactate removal apparently lagging behind the rate of lactate production [17]. The KSC whole-body model embraces seven tissues compartments or organs: brain, heart, liver, gastrointestinal tract, skeletal muscle, adipose tissue and a generic *other tissues*. Each compartment is connected to the others via blood circulation and is described by dynamic mass balance equations for 25 major cellular metabolic reactions involving a total of 22 substrates. A brief description of the KSC model is included in S2 Appendix in “Supporting information”. For what concerns the effect of physical exercise, the basic assumption of the model is that changes of epinephrine concentration in blood due to exercise affect the pancreatic release of glucagon and insulin [13].

**Fig 1:**
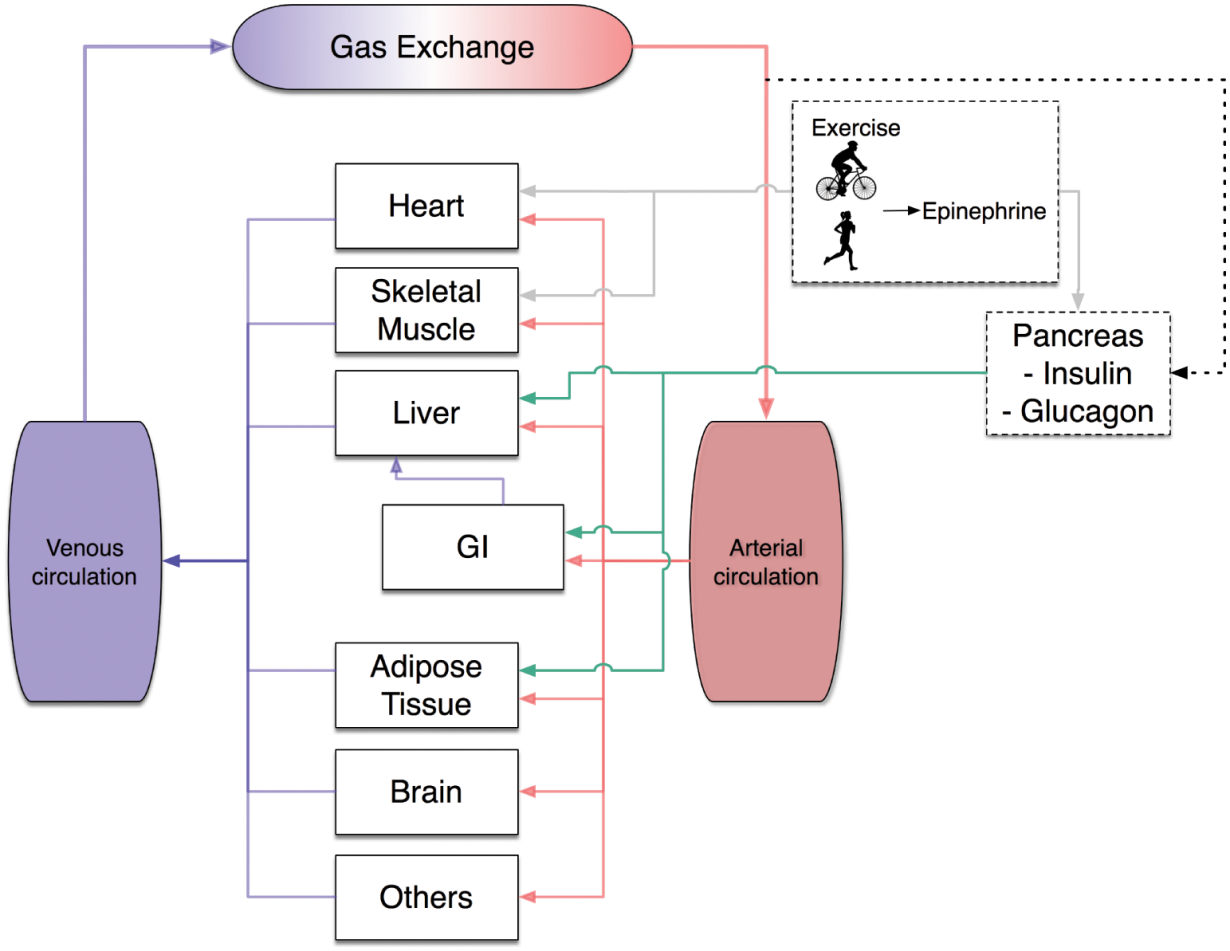
Whole body system diagram. Exercise stimulates epinephrine release which influences the pancreatic secretion of insulin and glucagon and acts as a neuroendocrine signal for the heart and skeletal muscle (gray lines). Consequently, modifications in the glucagon and insulin production modulate in a coordinated way the metabolic flux rates of the different organs. Each organ is connected via the arterial/venous circulation (red/blue lines). Arterial glucose concentration (dotted line) signals the pancreas to set the levels of insulin and glucagon, whose ratio is used by the liver, GI tract and the adipose tissue. Two more ODEs are added to the KSC to model the exercise-induced effect of epinephrine, for a total of 136 ODEs consisting in the multi-scale computational model.

The modifications introduced to the KSC model do not change the original basic hypothesis, namely that changes in epinephrine levels are dependent on exercise intensity and related to the pancreatic release of glucagon and insulin. The glucagon-to-insulin ratio modulates the metabolic fluxes of organs, as depicted in Fig 1.

In what follows we introduce the modifications made, namely: i) the use of the oxygen consumption in place of the WR (“relative” rather than “absolute” exercise intensity); ii) modeling how oxygen consumption relates to epinephrine; iii) explaining how the glucagon/insulin controller is modified by the new description of exercise.

The input parameters of the resulting multi-scale whole-body computational model here proposed are listed in Table 1.

**Table 1:** Parameters defining the subject performing physical exercise.

Parameter	
*Gender*	male/female
*Age*	age in years
*BW*	body weight in kg
T_v_	target value of exercise intensity (%VO_2max_)
$t_{ex}^{start}$, $t_{ex}^{end}$	start/end of the exercise session in min
*fitness status*	cardiorespiratory fitness classification from ‘poor’ to ‘superior’

The code has been completely written in ANSI C computer language to achieve maximal performance and portability. The differential equations are solved numerically with the CVODE library, a solver for stiff and nonstiff ordinary differential equation systems [18, 19].

### Linking the work rate to oxygen consumption

The maximal peak oxygen consumption (VO_2max_) was defined by Hill and Lupton in [20] as the maximal oxygen uptake attained despite further increases in exercise workload, thereby defining the limits of the cardiorespiratory system. VO_2max_ is widely recognized as the gold standard for determining cardiorespiratory fitness level, and sets the upper limits of the aerobic pathway [21]. For maximal model flexibility in predicting responses to different categories of subjects, VO_2max_ is determined on the basis of age, gender and fitness status as described by Heyward in [22]. The target value (T_v_) of exercise intensity in terms of percentage of VO_2max_ (T_v_ = %VO_2max_) [20] is used as input for the new model (in place of the work rate WR) and the steady state value for the oxygen consumption due to the exercise performed VO_2_ is computed as

$${\text{VO}}_{2}=\frac{T_{v}\cdot VO_{2max}}{100}.$$ (1)

The original KSC model specifies the WR in Watts. Since many metabolic fluxes and reactions depend on the parameter WR [13], we need to express WR as a function of VO_2_. To simulate different exercise modalities, we resorted to the metabolic equations provided by the American College of Sports Medicine (ACSM) [23] to estimate the oxygen consumption for five different exercise modalities. As for leg and arm cycling, the following linear equations relate WR (expressed in Watt, i.e., WR(W)) to the oxygen consumption during a *moderate-intensity* exercise:

$$\text{WR}\left(\text{W}\right)=\{\begin{array}{cc} BW\cdot (VO_{2}-2\cdot VO_{2,rest})/10.8 & \text{for}\;\text{leg}\;\text{cycling}\\ BW\cdot (VO_{2}-VO_{2,rest})/18 & \text{for}\;\text{arm}\;\text{cycling} \end{array}$$

where BW is the body weight, VO_2,rest_ is the O_2_ uptake at rest. For what concerns walking, running and stepping, the ACSM metabolic equations for the oxygen cost are:

$${\text{VO}}_{2}=\{\begin{array}{cc} VO_{2,rest}+0.1\cdot v+1.8\cdot v\cdot G & \text{for}\;\text{walking}\\ VO_{2,rest}+0.2\cdot v+0.9\cdot v\cdot G & \text{for}\;\text{running}\\ VO_{2,rest}+0.2\cdot F+1.33\cdot 1.8\cdot H\cdot F & \text{for}\;\text{stepping} \end{array}$$ (2)

in which *v* is speed expressed in m/min, *G* is the percent of slope expressed as a ratio, *F* is the stepping frequency expressed in steps/min and *H* is step height in m. To obtain the WR for the last three categories of exercise, the activity energy expenditure is computed as the milliliters of oxygen consumed during physical exercise converted to Watt [23]. Assuming a caloric equivalent for 1 liter of oxygen approximatively 5 kcal [24, 25], the following relationship is used to determine the Watt consumed per minutes for walking, running and stepping

$$\text{WR}\left(\text{W}\right)=VO_{2}\cdot BW\cdot \left(t_{ex}^{end}-t_{ex}^{start}\right)\cdot 5\cdot 10^{-3}\cdot 1.163\;\text{for}\;\text{walking,}\;\text{running,}\;\text{stepping}$$

in which $t_{ex}^{start}$ and $t_{ex}^{end}$ are the beginning and the end of the exercise session expressed in minutes. For walking, running and stepping, the target value T_v_ of exercise intensity is obtained from Eq (1) after computing VO_2_ from Eq (2).

### Modeling epinephrine dynamics versus oxygen consumption

The second improvement on the KSC model regards the description of the dynamics of plasma epinephrine. It directly results from the model of the oxygen consumption described in the previous section. The original formulation of the dynamics of epinephrine in the KSC model is in Eq (1) of the S2 Appendix in “Supporting information”.

To account for the exercise variability, a set of differential equations describing the dynamics of epinephrine is implemented, in which the dependence on exercise intensity is explicitly given. The novelty in the new description of the exercise consists in modeling the dynamics of oxygen uptake (VO_2_) on the basis of the work by Roy and Parker [12], as already done in a previous work by our group [26]. Changes in oxygen consumption during the exercise session and the subsequent recovery phase are described in terms of %VO_2max_ by means of the following linear first-order differential equation:

$$\frac{{\text{dPVO}}_{2\text{max}}\left(\text{t}\right)}{\text{dt}}=-0.8\cdot PVO_{2max}\left(t\right)+0.8\cdot u\left(t\right)$$ (3)

in which *PVO*_2*max*_(*t*) is the suprabasal oxygen consumption, expressed as a percentage of the maximum value (%VO_2max_) and *u*(*t*) describes the input as a step function assuming value T_v_ for the entire duration of the exercise, that is

$$\text{u}\left(\text{t}\right)=\{\begin{array}{cc} 0 & 0\leq t<t_{ex}^{start}\\ T_{v} & t_{ex}^{start}\leq t\leq t_{ex}^{end}\\ 0 & t>t_{ex}^{end}. \end{array}$$

In Eq (3) the coefficient 0.8 min^-1^ is chosen to allow *PVO*_2*max*_ to reach T_v_ in approximately 5-6 minutes after the beginning of the exercise, as proposed by Roy and Parker [12]; $t_{ex}^{start}$ and $t_{ex}^{end}$ refer to the begin and the end of the session of physical exercise. After reaching T_v_, the oxygen consumption remains constant for the duration of the exercise and then returns to its basal value in 5-6 minutes after the end of the exercise according to a first order dynamics.

The model of epinephrine secretion and elimination (whose inputs are based on the %VO_2max_ proposed by Kildegaard and colleagues in [14]) is adapted to describe the changes in epinephrine concentration due to exercise as follows

$$\frac{{\text{dC}}_{\text{E}}\left(\text{t}\right)}{\text{dt}}=\frac{1}{V_{d}}\cdot \left(f_{1}+f_{2}+f_{3}\right)\cdot BW-k\cdot C_{E}\left(t\right)$$ (4)

in which *C*_*E*_(*t*) is the epinephrine concentration, *V*_*d*_ is the volume of distribution, *f*_1_ is a constant representing a basal epinephrine secretion, *k* is the epinephrine elimination constant. The term *f*_2_ accounts for the epinephrine contribution depending on the arterial glucose level (expressed by *Ca*, *g*(*t*)) and is described by

$${\text{f}}_{2}=f_{2}\left(C_{a,g}\left(t\right)\right)=c_{1}/(1+e^{c_{2}\cdot \left(Ca,g\left(t\right)-c_{3}\right)})$$ (5)

and *f*_3_ is the contribution from the physical activity, depending on its relative intensity

$${\text{f}}_{3}=f_{3}\left(PVO_{2max}\left(t\right)\right)=d_{1}/(1+e^{d_{2}\cdot \left(d_{3}-PVO_{2max}\left(t\right)\right)}).$$ (6)

In Eq (6), PVO_2max_(t) represents the actual level of T_v_, as described in Eq (3). The epinephrine elimination constant *k* is computed by imposing the steady-state condition in Eq (4). Consequently, Eqs (5) and (6) are calculated in the absence of physical activity (i.e., *T*_*v*_ = 0, *PVO*_2*max*_(*t*) = 0) and with the arterial glucose concentration corresponding to the fasting value $C_{a,g}^{*}$ of 5 mmol/l, thus obtaining

$${\text{f}}_{2}^{*}=f_{2}\left(C_{a,g}^{*}\right)=c_{1}/(1+e^{c_{2}\cdot \left(C{a,g}^{*}-c_{3}\right)})$$

and

$${\text{f}}_{3}^{*}=f_{3}\left(0\right)=d_{1}/(1+e^{d_{2}\cdot d_{3}}).$$

Then, the elimination constant *k* is computed by imposing in Eq (4), no changes in the epinephrine concentration, namely with the epinephrine level corresponding to the basal value *C*_*E*,0_

$$\text{k}=\frac{BW}{V_{d}\cdot C_{E,0}}\cdot \left(f_{1}+f_{2}^{*}+f_{3}^{*}\right).$$

### Modifications to the hormonal glucagon/insulin model

The multi-scale KSC model of glucose homeostasis incorporates the hormonal model by Saunders et al., in which both glucagon and insulin are produced and glucose regulation is achieved by altering the balance between the two hormones [27]. The original equations of the KSC hormonal model are included in the Eq (2) of the S2 Appendix in “Supporting information”. In this work, the original KSC model has been slightly modified yet remaining in accordance with the study of Saunders. Namely, the epinephrine contribution to the insulin dynamics is included in the same way as the terms describing glucagon and insulin itself, that is

$$\frac{{\text{dC}}_{\text{I}}}{\text{dt}}=C_{I}\left(t\right)\cdot [\psi \cdot [h-k_{3}\cdot \left(C_{G}\left(t\right)-C_{G,0}\right)-k_{4}\cdot \left(C_{I}\left(t\right)-C_{I,0}\right)-k_{5}\cdot \left(C_{E}\left(t\right)-C_{E,0}\right)]-D]$$ (7)

with the following equation for the glucagon

$$\frac{{\text{dC}}_{\text{G}}}{\text{dt}}=C_{G}\left(t\right)\cdot [\phi \cdot [h-k_{1}\cdot \left(C_{G}\left(t\right)-C_{G,0}\right)-k_{2}\cdot \left(C_{I}\left(t\right)-C_{I,0}\right)]-D]$$

in which *C*_*I*_(*t*) and *C*_*G*_(*t*) are the insulin and the glucagon blood concentrations, *C*_*I*, 0_, *C*_*G*, 0_ and *C*_*E*, 0_ are their basal values.

Since we have modified the epinephrine contribution to the insulin dynamics, we had to choose a new value for the parameter *k*_5_. To find the optimal value for *k*_5_ we have used a weighted non-linear least squares approach based on the Levenberg-Marquardt algorithm implemented in the *lsqnonlin* MATLAB (The MathWorks, Natick, MA, USA) function. The differential equations of the model are solved using *ode15s* MATLAB function, an implicit integration algorithm for stiff systems of equations. The parameter *D* is set to 0.1 and *h* is computed by imposing the steady-state condition with blood glucose concentration of 5 mmol/l, as in the original KSC model [13] and according to Saunders et al. [27]. Also the basal values *C*_*I*, 0_, *C*_*G*, 0_, *C*_*E*, 0_, the parameters *k*_1_, *k*_2_, *k*_3_, *k*_4_ and the functions *ϕ* and *ψ* are taken from the original KSC model. The parameter *k*_5_, along with its *CV*%, is estimated as in [13] by fitting plasma insulin and glucagon concentrations mean data obtained during an exercise session of 60 min. Data is collected by Hirsch and colleagues [28] from the mean of the experimental data obtained from thirteen normal young men before, during and after 60 min of exercise at T_v_ = 60 (see Table 2 for further details). The errors in the glucagon and insulin measurements are assumed to be normally distributed random variables, with zero mean and a constant percent coefficient of variation equal to 4%. We assumed 4% as a reasonable value accounting for mean inter- and intra-assay coefficient of variation (*CV*) for glucagon and insulin measurements, since no information was reported in the study by Hirsch. The precision of the estimate of parameter *k*_5_ is expressed using the percent coefficient of variation, *CV*% = (*SDe*/*e*), where the standard deviation *SDe* is derived from the inverse of the Fisher information matrix and *e* is the corresponding parameter estimate [29].

**Table 2:** Experimental data sets used for model validation.

Study	Subjects’ characteristics				Exercise parameters			Observables	
	Sex	Age	BW	Height	E_D_	T_v_	VO_2max_	Hormones	Substrates
1	6/0	28-30	70-78	1.80-1.88	8x4	38,51,64,77	35	GLUCA^‡^	GLU^‡^
[30]	6/0	25-27	65-73	1.72-1.78	8x4	38,51,64,77	50	EPI^‡^	GLR^‡^
								INS^‡^	LAC^‡^
									FFA^‡^
									ALA^‡^
2	8/0	23-40	71-89	1.74-1.88	40	60	46	GLUCA^‡^	GLU^‡^
[32]									GLR^‡^
									LAC^‡^
									FFA^‡^
3	13/0	22-28	66-85	1.80*	60	60	44	INS^‡^	FFA^‡^
[28]								GLUCA^‡^	
								EPI^‡^	
4	7/0	21-23	69-82	1.80*	120	40	39		LAC^‡^
[33]		25-27	69-82	1.80*	120	40	58		
5	8/0	21-23	76-81	1.73-1.79	90	60	42	EPI^‡^	GLR^‡^
[34]	0/8	21-23	64-70	1.64-1.66	90	60	32		LAC^‡^
									FFA^‡^
6	13/0	20-30	64-84	1.69-1.93	180	31	61		GLY^¤^
[35]					120	64	61		

Sex is m/f; Age is in years; Body Weight is in kg; Height is in m; E_D_ = exercise duration in min; T_v_ = exercise intensity in %VO_2max_; VO_2max_ is in ml ⋅ kg^-1^ ⋅ min^-1^.

GLUCA: glucagon; INS: insulin; EPI: epinephrine; FFA: fatty free acids; LAC: lactate; GLU: glucose; GLR: glycerol, ALA: alanine; GLY: glycogen; PCR: phosphocreatine.

^‡^ Refers to the plasma content.

^¤^ Refers to the muscular content.

* Refers to an arbitrary imposed value, since not explicitly given in the study.

The study numbers refer to the list of the works mentioned above.

### Model validation

The refined model of the effects of a session of exercise on whole body metabolism has then been validated against experimental data from independent studies, with different exercise modalities and individuals’ characteristics. We referred to the following studies.

1. **Bloom** et al. [30]: well-trained and untrained volunteers were studied during and immediately after four successive 7 minutes periods of exercise at 30, 45, 60 and 75% of their maximal work capacity (W_max_). Since our computational model uses %VO_2max_ as input parameter, a conversion from W_max_ to VO_2max_ is made taking into consideration the same relationship between oxygen consumption during moderate-intensity exercise and WR reported in the study by Arts and Kuipers [31]. As a consequence 30, 45, 60 and 75 %W_max_ correspond to T_v_ = 38, 51, 64 and 77. Blood samples were collected at rest, at the end of each exercise period and 5 min following the end of exercise, for estimation of metabolites in blood.
2. **Wahren** et al. [32]: arterial concentrations of substrates and glucagon were measured in male individuals at rest and during 40 minutes of exercise performed at T_v_ = 60.
3. **Hirsch** et al. [28]: the normal exercise-associated changes in plasma concentration of insulin, glucagon, epinephrine and FFA were analyzed in young men. Baseline observations started 30 min before the exercise. Exercise was performed at T_v_ = 60 for 60 min; observations continued for 120 minutes in the following recovery period [28]. Experimental data for insulin and glucagon is used for parameter estimation, whereas the model simulations of epinephrine and FFA dynamics are here reported for model validation.
4. **Bergman** et al. [33]: blood samples were collected in trained and untrained men while they were performing a moderate-intensity exercise below the LT (indicated as 71%VO_2max_ for trained subjects in the study). Blood samples were drawn at rest (0 min), and at 15, 30, 45, 60, 90 and 120 min during exercise.
5. **Carter** et al. [34]: male and female volunteers exercised for 90 min at an intensity of T_v_ = 60. Blood samples were drawn at rest (0 min), and at 30, 60, 75, and 90 min during exercise.
6. **Gollnick** et al. [35]: subjects exercised for 180 min at a low-intensity of T_v_ = 31 and for 120 min at a moderate-intensity of T_v_ = 64. Measures of muscle glycogen were obtained at 0, 40, 120 and 180 min for the low-intensity exercise and at 0, 20, 60 and 120 min for the moderate-intensity exercise.

All the exercise protocols analyzed were performed by healthy individuals in fasted condition, exercising under the LT on a cycle ergometer. The subjects considered in these studies differ in gender, physical fitness status and anthropometric measures. It is important to notice that, although some of these studies were used by Kim and colleagues to validate their model, they limited the validation to only one exercise condition (i.e., WR fixed to 125W and power output at T_v_ = 60 for 60 min) and one kind of subject (a 70kg untrained male). Further details for each study are reported in Table 2.

## Results

### Parameter estimation

**Fig 2:**
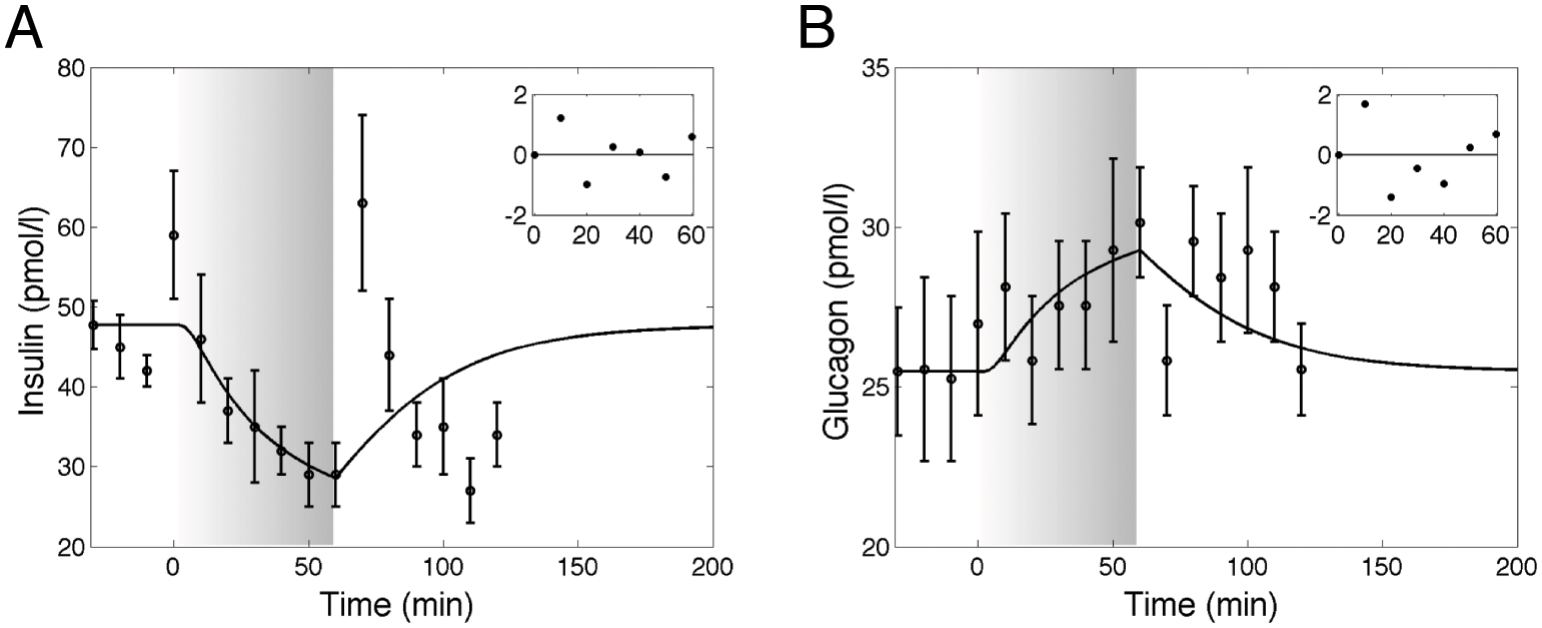
Dynamics of insulin and glucagon in response to exercise. Model fit (solid line) vs experimental data (circles), expressed as mean ± SEM from study 3 [28]. Parameter estimation is performed using data obtained during an exercise session of 60 min. The gray zone refers to the exercise period. The inset plots refer to the weighted residuals. A: Plasma insulin concentration. B: Plasma glucagon concentration.

The computational model here proposed simulates the responses of hormones and metabolites during a session of moderate intensity aerobic exercise for different typologies of subjects and physical exercise performed. To allow this diversity, we have modified the hormonal glucagon/insulin model and we have estimated again the parameters. The value resulting from parameter estimation is *k*_5_ = 3.6 ⋅ 10^−5^ *pM*^−1^ ⋅ *min*^−1^ and the related *CV*% is 3.9. Results of the estimation procedure on the mean of the experimental data obtained from thirteen normal young men studied from Hirsch et al. [28] are reported in Fig 2 and the plots of the time course of the weighted residuals are shown in the inset plots.

### Whole-body arterial concentrations of hormones and metabolites

**Fig 3:**
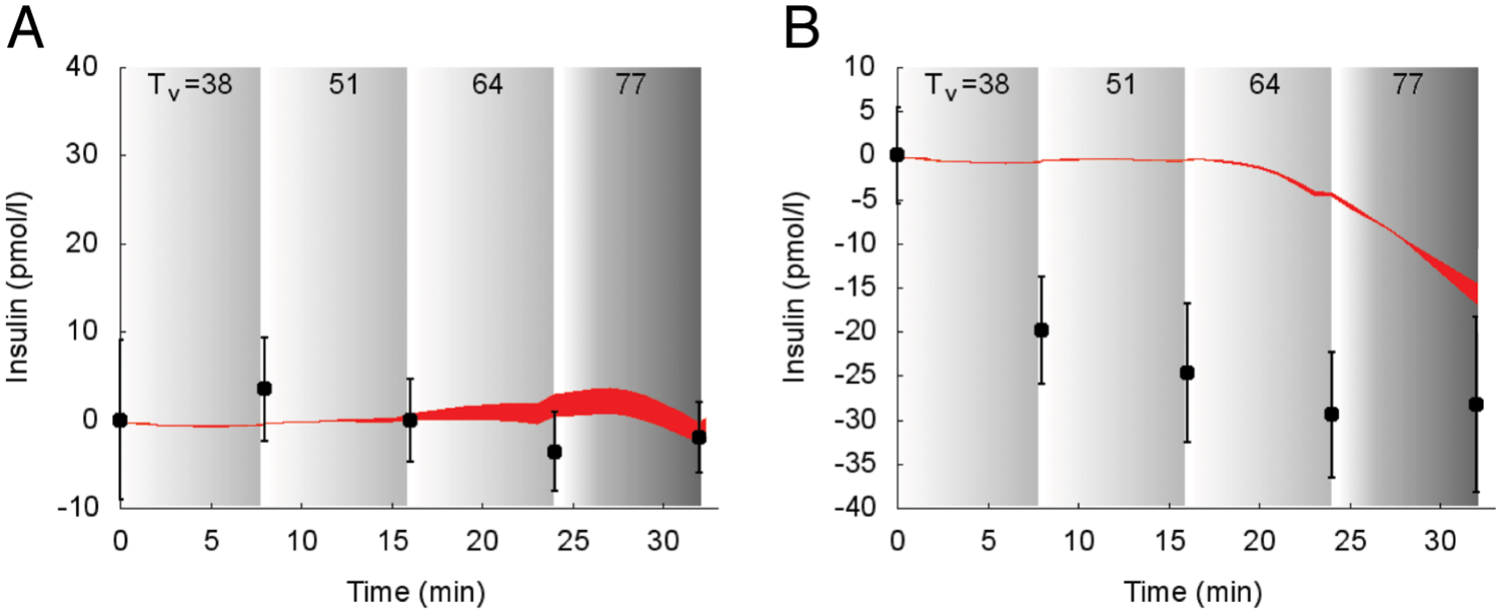
Dynamics of suprabasal plasma insulin concentration in response to exercise. Model fit (red areas) vs experimental data (circles) expressed as mean ± SEM from the study 1. The red areas show the range of the dynamic responses of the model to the variability of the subjects’ characteristics as reported in Table 2; individual response curves related to each simulation are available. The gray zone refers to the exercise period. The dark gray zone refers to the exercise performed above the LT until minute 32. A: Trained individuals. B: Untrained individuals.

Model predictions of suprabasal **insulin** dynamics superimposed to experimental data is reported in Fig 3 for study 1 [30].

The simulation overlaps the measured values for trained subjects (Panel A). Measured insulin concentrations show a decrease during exercise in untrained subjects (Panel B) and model-predicted insulin concentration shows a consistent decrease, although it appeared slightly delayed.

**Fig 4:**
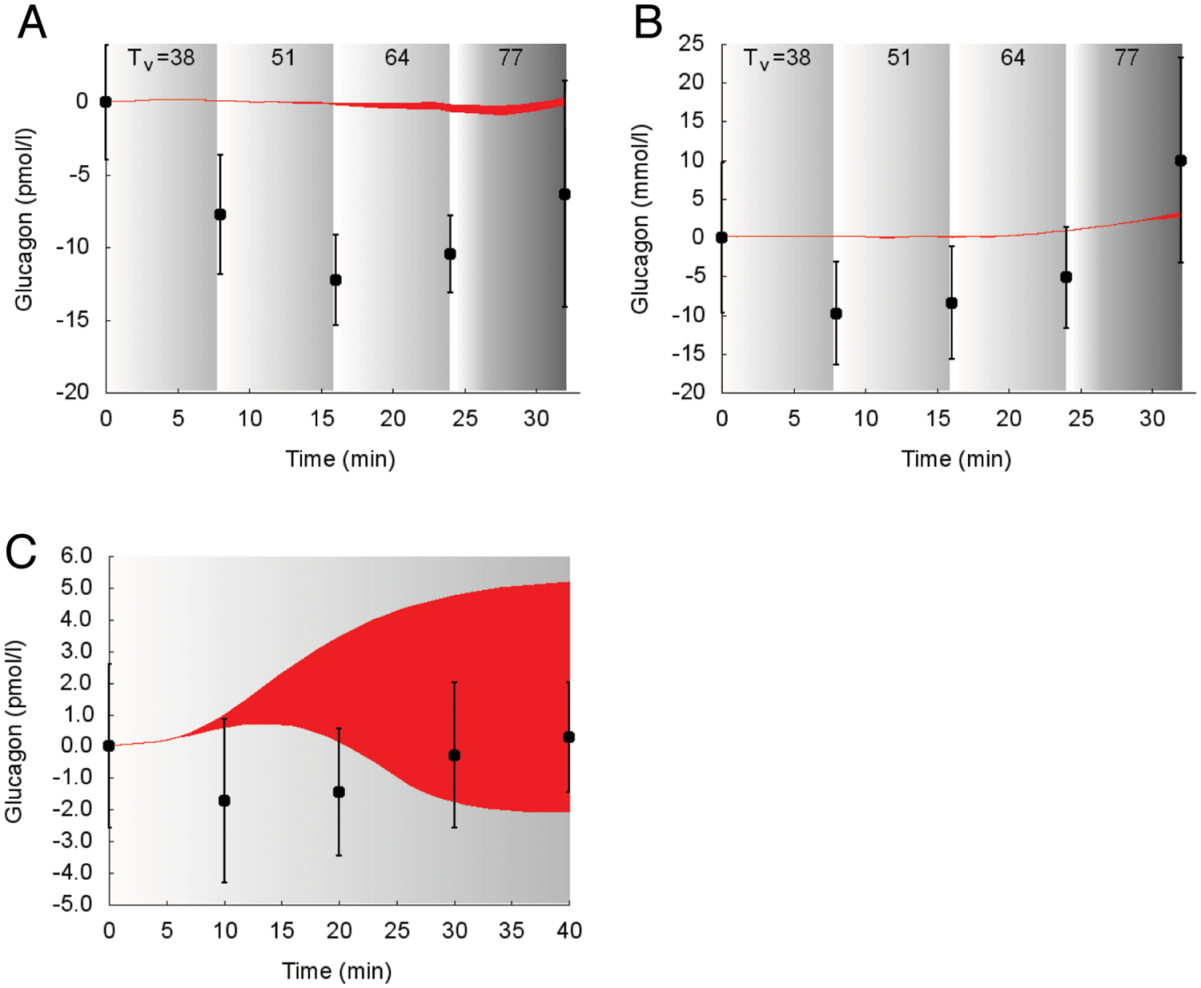
Dynamics of suprabasal plasma glucagon concentration in response to exercise. Model fit (red areas) vs experimental data (circles), expressed as mean ± SEM. The red areas show the range of the dynamic responses of the model to the variability of the subjects’ characteristics as reported in Table 2; individual response curves related to each simulation are available. The gray zone refers to the exercise period. The dark gray zone refers to the exercise performed above the LT until minute 32. A: Trained individuals from the study 1. B: Untrained individuals from the study 1. C: Individuals from the study 2.

In Fig 4 the suprabasal dynamics of **glucagon** superimposed to experimental data is shown for the protocols reported in the studies 1, 2 [30, 32].

The simulated glucagon response to incremental exercise was not accurately following the data produced by Bloom et al., whereas it shows a good agreement with data from the study by Wahren.

**Fig 5:**
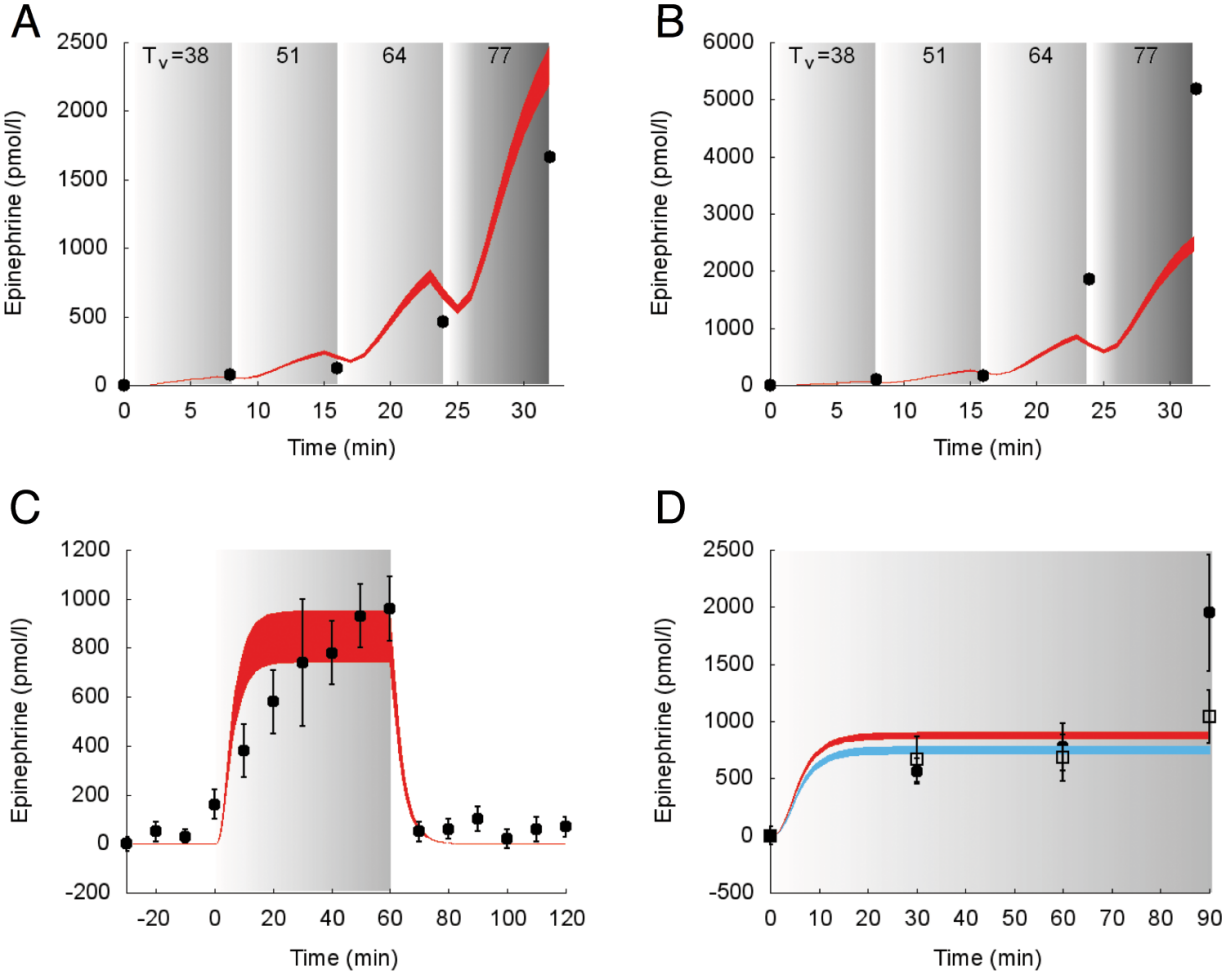
Dynamics of suprabasal plasma epinephrine concentration in response to exercise. Model fit (colored areas) vs experimental data (circles and squares) expressed as mean ± SE. The colored areas show the range of the dynamic responses of the model to the variability of the subjects’ characteristics as reported in Table 2 (black circles and red area: males; open squares and blue area: females); individual response curves related to each simulation are available. The gray zone refers to the exercise period. The dark gray zone refers to the exercise performed above the LT until minute 32. A: Trained individuals from the study 1. B: Untrained individuals from the study 1. C: Individuals from the study 3. D: Individuals from the study 5 (black circles and red area: males; open squares and blue area: females).

The model predictions of the suprabasal dynamics of **epinephrine** superimposed to experimental data for the protocols reported in the studies 1, 3 and 5 [28, 30, 34] are shown in Fig 5.

Similarly to experimental data, the simulated suprabasal epinephrine concentration increases at the onset of the exercise, remains nearly constant during exercise and then decreases at the end of exercise. During incremental exercise below the LT (e.g., in the Bloom protocol [30]), model-predicted epinephrine and experimental data overlap in trained individuals (panel A). Conversely, epinephrine model response in untrained individuals is slightly underestimated (panel B).

**Fig 6:**
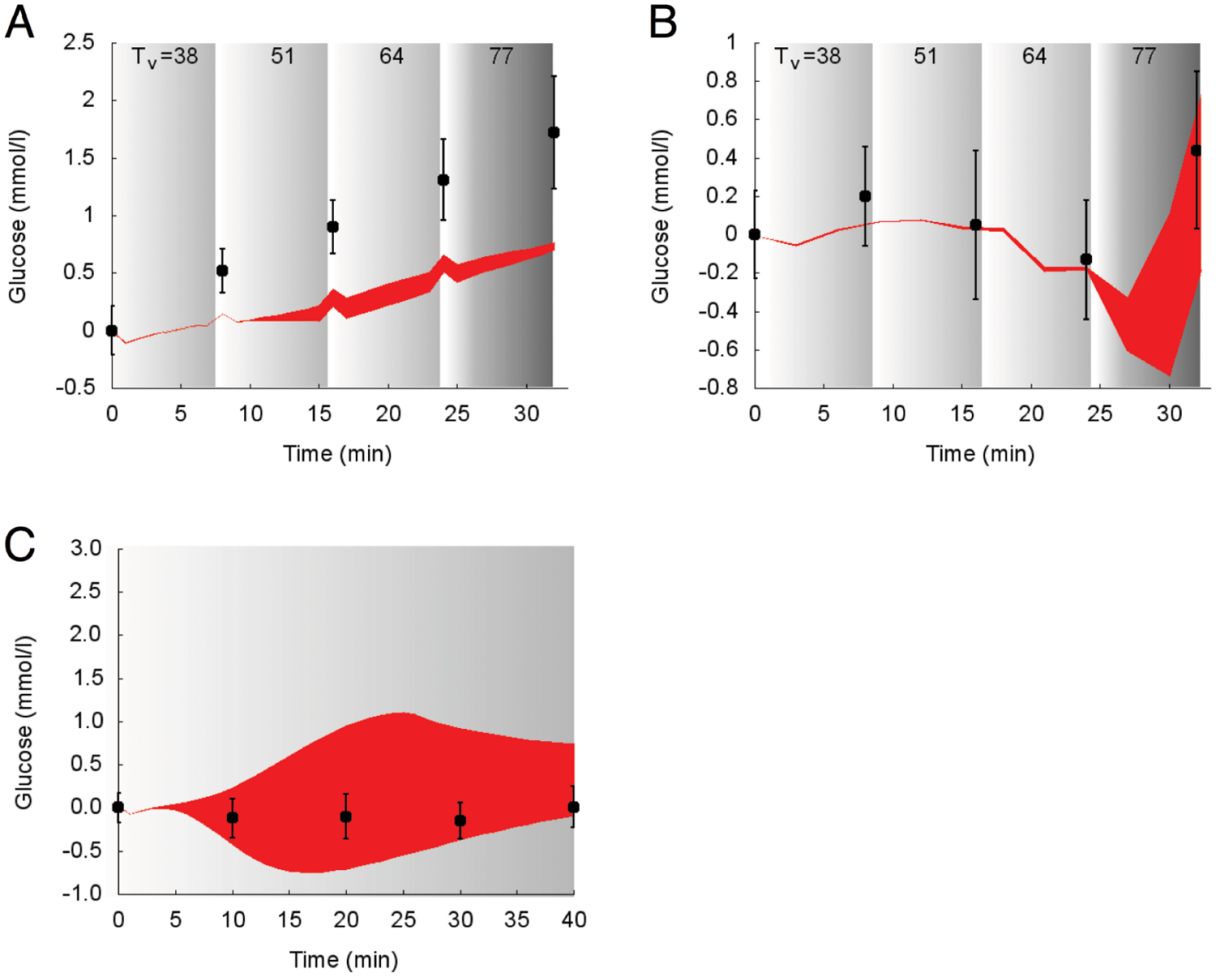
Dynamics of suprabasal plasma glucose concentration in response to exercise. Model fit (red areas) vs experimental data (circles) expressed as mean ± SE. The red areas show the range of the dynamic responses of the model to the variability of the subjects’ characteristics as reported in Table 2; individual response curves related to each simulation are available. The gray zone refers to the exercise period. The dark gray zone refers to the exercise performed above the LT until minute 32. A: Trained individuals from the study 1. B: Untrained individuals from the study 1. C: Individuals from the study 2.

Model predictions of the suprabasal dynamics of **glucose** superimposed to experimental data for the protocols reported in the studies 1 and 2 [30, 32] are shown in Fig 6.

During incremental exercise below the LT (e.g., in the Bloom protocol [30]), simulated glucose concentration and experimental data overlap for trained individuals (panel A), whereas the simulated concentration of glucose is slightly underestimated in trained individuals (panel B). In panel C, the deviation from the experimental data is physiologically consistent.

**Fig 7:**
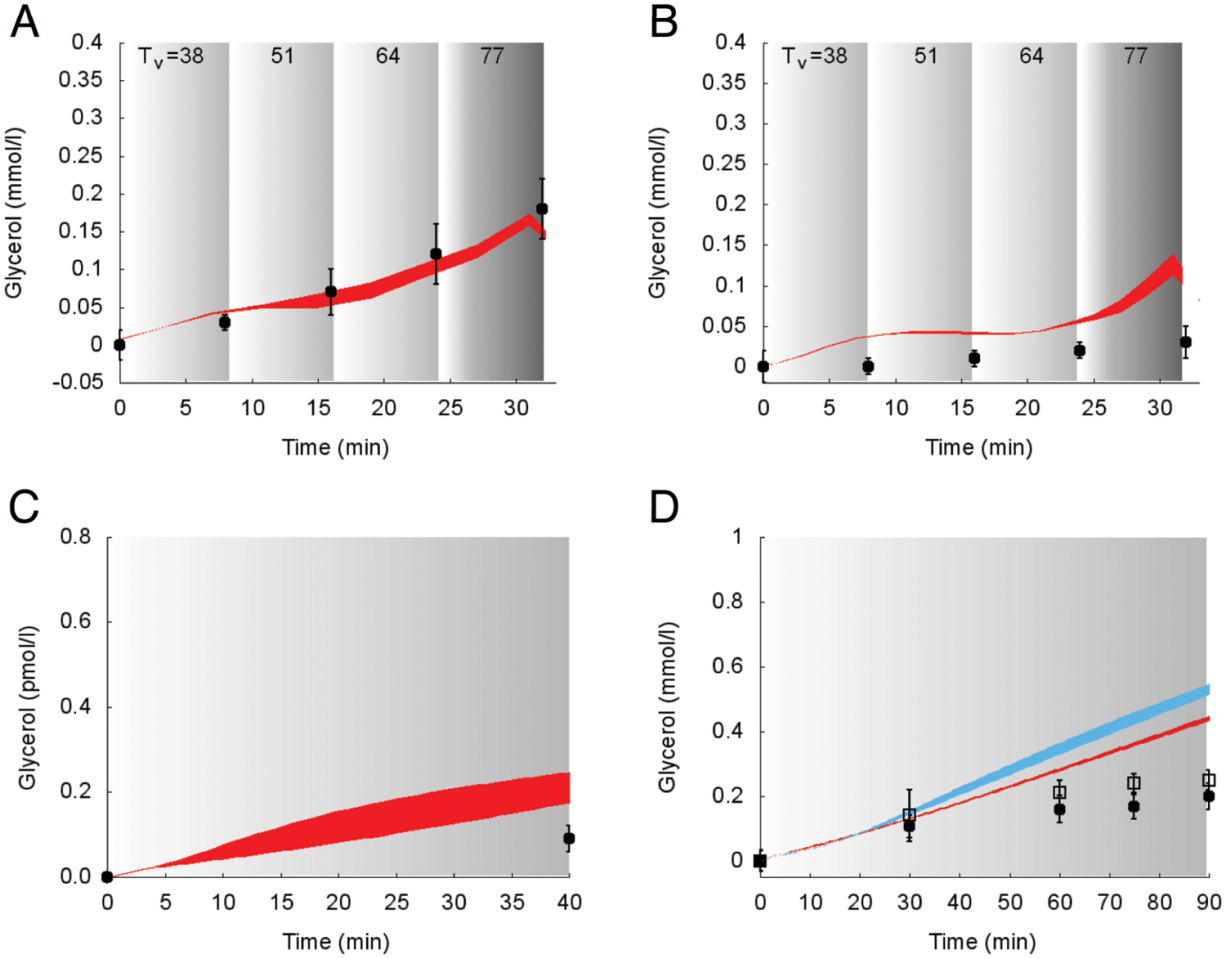
Dynamics of suprabasal plasma glycerol concentration in response to exercise. Model fit (colored areas) vs experimental data (circles and squares) expressed as mean ± SE. The colored areas show the range of the dynamic responses of the model to the variability of the subjects’ characteristics as reported in Table 2 (black circles and red area: males; open squares and blue area: females); individual response curves related to each simulation are available. The gray zone refers to the exercise period. The dark gray zone refers to the exercise performed above the LT until minute 32. A: Trained individuals from the study 1. B: Untrained individuals from the study 1. C: Individuals from the study 2. D: Individuals from the study 5.

**Fig 8:**
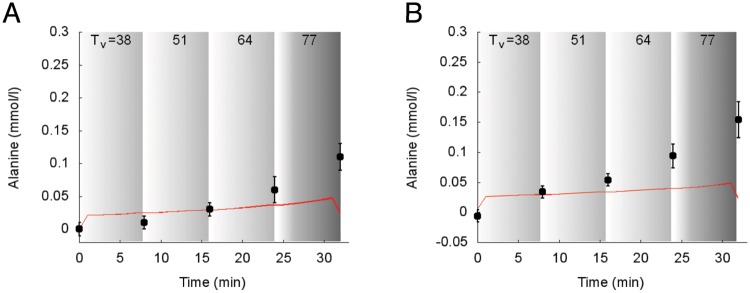
Dynamics of suprabasal plasma alanine concentration in response to exercise. Model fit (red areas) vs experimental data (circles) expressed as mean ± SE from the study 1. The red areas show the range of the dynamic responses of the model to the variability of the subjects’ characteristics as reported in Table 2; individual response curves related to each simulation are available. The gray zone refers to the exercise period. The dark gray zone refers to the exercise performed above the LT until minute 32. A: Trained individuals. B: Untrained individuals.

Simulations of the suprabasal dynamics of **glycerol** and **alanine** superimposed to experimental data are shown in Figs 7 and 8 for the studies 1, 2 and 5 [30, 32, 34].

Model simulations and experimental values show similar dynamics in all the considered protocols. The model slightly overestimates and underestimates, respectively, the values of glycerol and alanine plasma concentrations.

**Fig 9:**
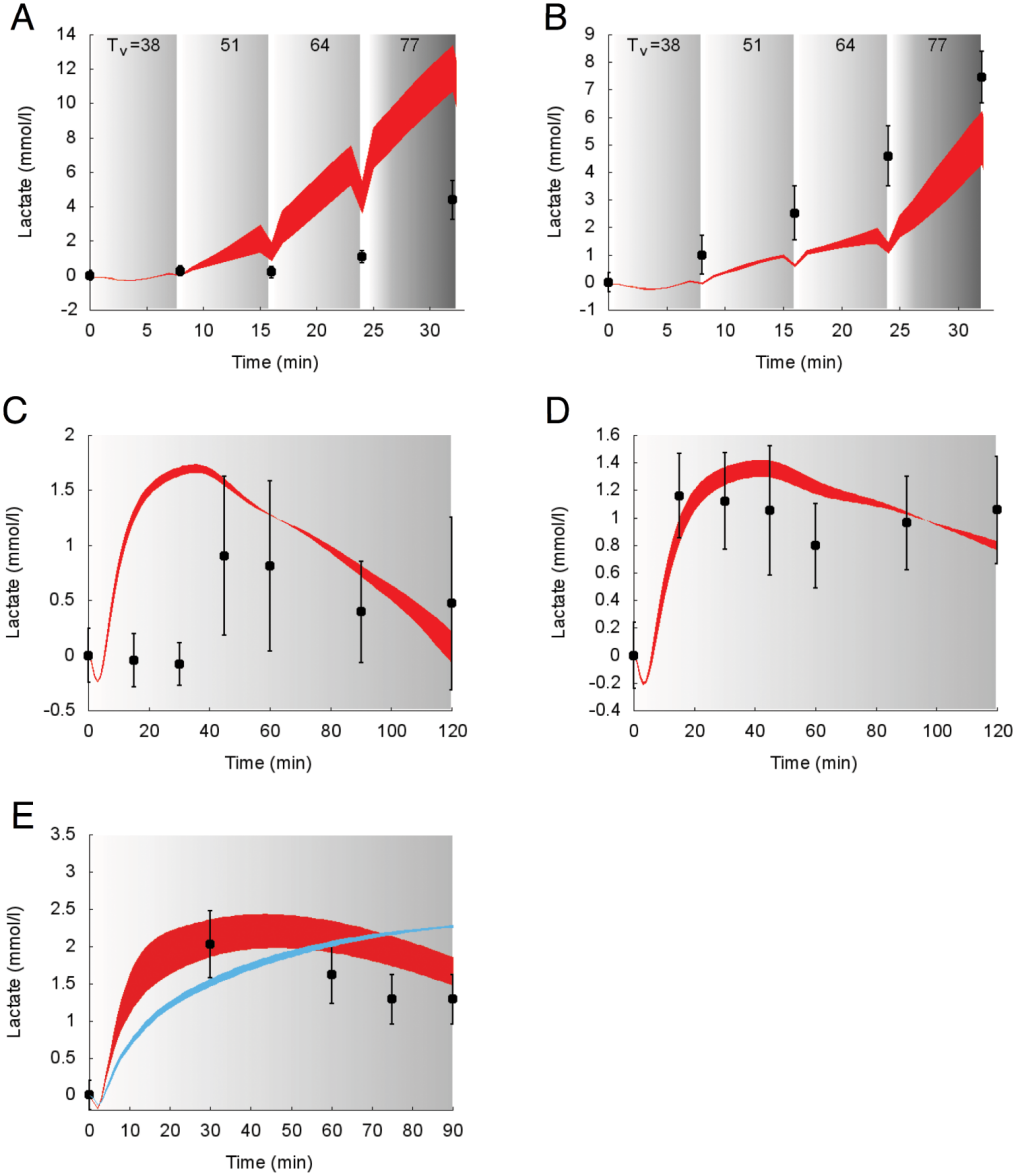
Dynamics of suprabasal plasma lactate concentration in response to exercise. Model fit (colored areas) vs experimental data (circles) expressed as mean ± SE. The colored areas show the range of the dynamic responses of the model to the variability of the subjects’ characteristics as reported in Table 2 (blue: females; red: males); individual response curves related to each simulation are available. The gray zone refers to the exercise period. The dark gray zone refers to the exercise performed above the LT until minute 32. The colored area shows the dynamic responses of the model to the variability of the subjects’ characteristics as reported in Table 2. A: Trained individuals from the study 1. B: Untrained individuals from the study 1. C: Trained individuals from the study 4. D: Untrained individuals from the study 4. E: Individuals from the study 5.

Suprabasal dynamics of **lactate** superimposed to experimental data for the protocols reported in the studies 1, 4 and 5 [30, 33, 34] are shown in Fig 9.

Model prediction and measured values during incremental exercise (e.g., in the work by Bloom and colleagues [30]) show similar behaviors, although the simulations slightly overestimate and underestimate the lactate concentrations for trained (panel A) and untrained individuals (panel B), respectively. With respect to the Bergman study [33] the model predicted lactate in panel C (trained subjects) shows an early increase similar to the one reported in panel D (untrained subjects), although the experimental data shows a delayed increase. In panel E, two different simulations are shown for male and female subjects; conversely the experimental data provided do not refer to a specific gender. Both simulations for males and females well fit the experimental data, with a better precision for male subjects.

**Fig 10:**
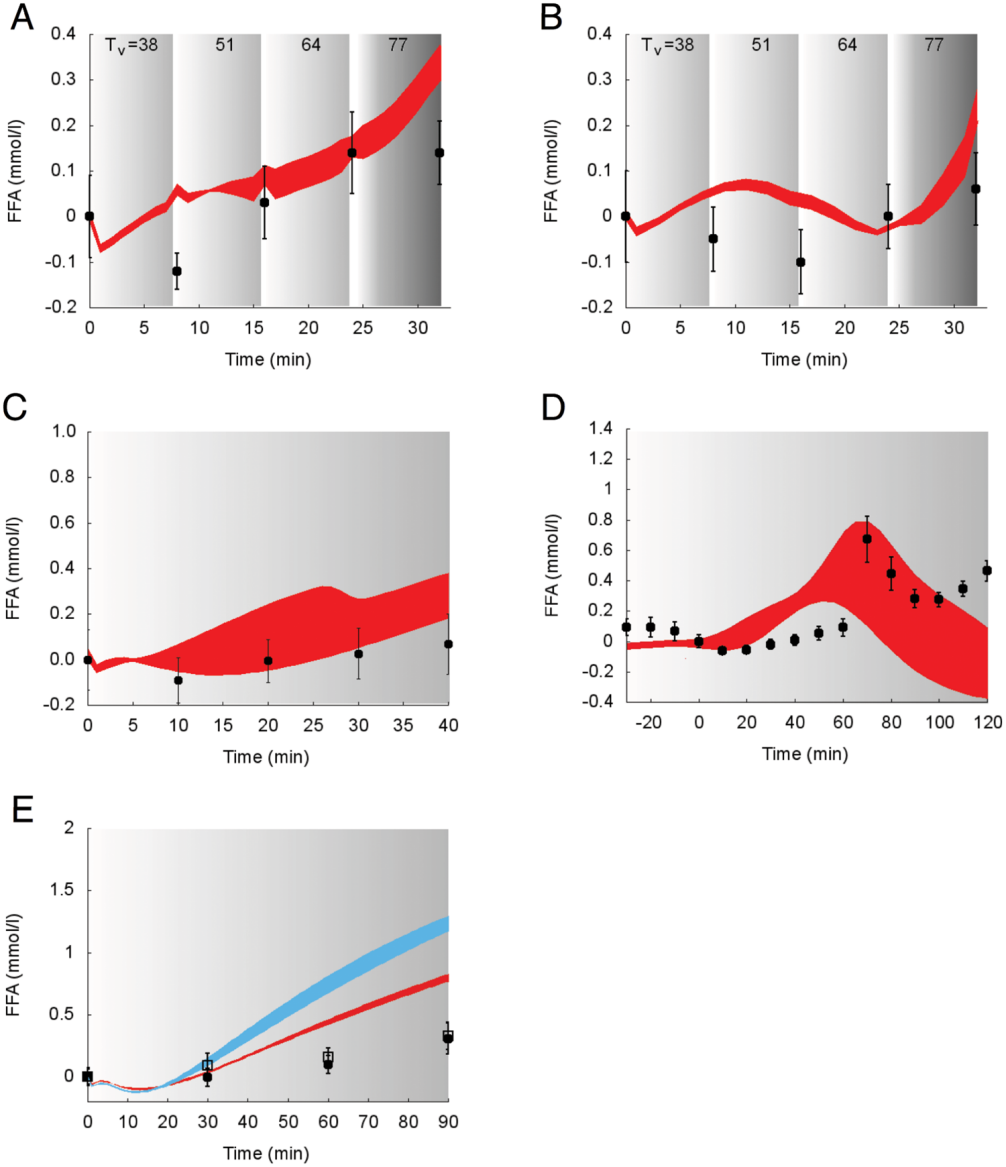
Dynamics of suprabasal plasma FFA concentration in response to exercise. Model fit (colored areas) vs experimental data (circles) expressed as mean ± SE. The colored areas show the range of the dynamic responses of the model to the variability of the subjects’ characteristics as reported in Table 2 (black circles and red area: males; open squares and blue area: females); individual response curves related to each simulation are available. The gray zone refers to the exercise period. The dark gray zone refers to the exercise performed above the LT until minute 32. A: Trained individuals from the study 1. B: Untrained individuals from the study 1. C: Individuals from the study 2. D: Individuals from the study 3. E: Individuals from the study 5.

Model predictions of the suprabasal dynamics of **FFA** superimposed to experimental data for the protocols performed in the studies 1, 2, 3 and 5 [28, 30, 32, 34] are reported in Fig 10.

In all the experimental data sets taken into consideration for model validation, a decreasing trend is evident at the onset of the exercise, followed by an increasing trend. Simulations and measured concentrations of FFA show similar fluctuations during exercise and a similar trend at the end of exercise, as evident in the recovery phase reported in Panel D.

### Reactions and fluxes of substrates in organs

**Fig 11:**
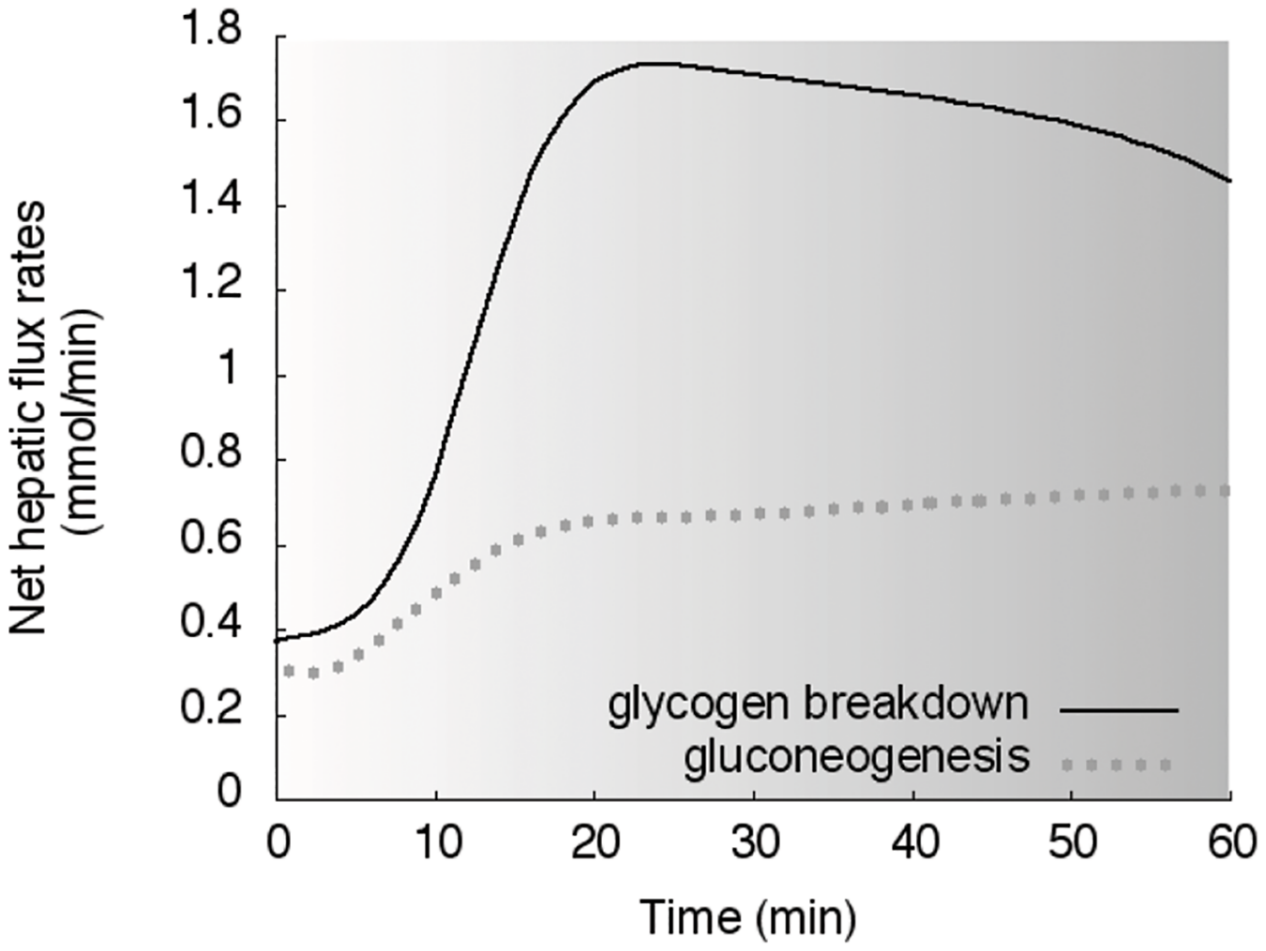
Reactions in liver. Simulations of net hepatic glycogen breakdown (solid line) and net hepatic gluconeogenesis (dotted line) in response to the exercise performed in the study 3. The gray zone refers to the exercise period.

The dynamic responses of net **hepatic** glycogenolysis and gluconeogenesis for the subjects in the study 3 [28] are shown in Fig 11.

**Fig 12:**
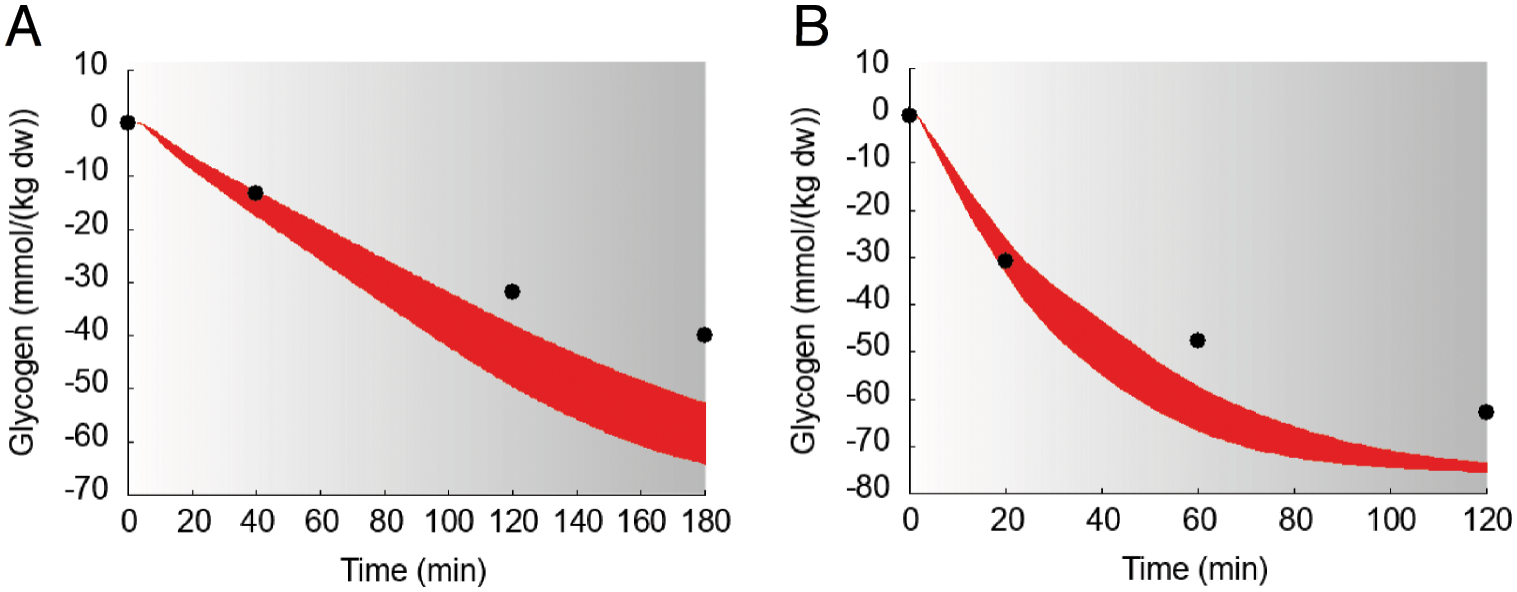
Dynamics of suprabasal concentration of glycogen in muscle in response to exercise. Model fit (red areas) vs experimental data (circles) expressed as mean ± SE in the study 6. The gray zone refers to the exercise period. The red areas show the range of the dynamic responses of the model to the variability of the subjects’ characteristics as reported in Table 2; individual response curves related to each simulation are available.

The dynamic responses of **muscular** content of glycogen for the subjects in the study 6 [35] are shown in Fig 12.

## Discussion

### Model development

In this study we proposed an extended formulation of the multi-scale computational model from Kim et al. [13], able to effectively describe fuel homeostasis in response to an exercise bout. This new formulation allows to model exercises differing in duration, modality, intensity (yet below the LT) performed by healthy subjects differing in gender, age, anthropometric characteristics and fitness status. In the original formulation of the KSC model, metabolic fluxes in the various tissues were hypothesized to be modulated in response to changes in the concentration of the hormone epinephrine, elicited by the exercise effort, expressed in terms of WR. In the KSC model a given value of WR (fixed to 125W power output at 60%VO_2max_) was used as the input parameter setting the exercise intensity.

However, this represents an absolute exercise intensity, and it is known that fixed absolute external workloads elicit heterogeneous levels of cardiovascular and metabolic stress in individuals with different fitness status and exercise capacity. Therefore, setting relative rather than absolute exercise intensity is preferred, as it allows a better characterization of the physiological acute and chronic responses to the exercise stimulus. This is of paramount importance for the investigation of exercise metabolism as well as for exercise prescription (and, in the case of the study, for a correct simulation of the exercise responses).

Relative exercise intensity can be monitored and controlled by using different parameters, such as percentages of maximal oxygen consumption (%VO_2max_) or heart rate (%HR_max_, %HRR) and metabolic thresholds, among others. VO_2max_ represents a universally accepted index of cardiorespiratory (aerobic) fitness; it is associated to the risk of mortality and for metabolic diseases, and provides important information on exercise capacity. Moreover, it can be accurately measured in a laboratory settings, but also estimated or assumed from normative values [23]. Therefore, we chose to implement our model with %VO_2max_, which is the most common method used to normalize the metabolic stress in the studies dealing with exercise metabolism in healthy and clinical populations thus allowing an appropriate validation with existing experimental data, otherwise problematic when considering absolute external workloads.

We acknowledge, however, that the use of this method for setting the relative intensity has been criticized by some authors who suggested a non-optimal standardization of metabolic stress based on the inter-individual variability in blood lactate response to a fixed %VO_2max_ [36, 37]. Consequently, the use of metabolic thresholds (lactate, aerobic and anaerobic threshold) has been proposed as more appropriate anchors for relative exercise intensity and therefore metabolic stress. However, also the consideration of these parameters (or the threshold concept) is not without limitations and criticisms; it cannot be easily predicted and therefore requires direct tests ideally involving multiple visits. In addition, several terminologies have been used interchangeably in the literature to describe the concept of metabolic thresholds, often referring to phenomena occurring at different metabolic intensities with the same name [38]. In view of these facts, for the purpose of validating our proposed model with the existing data, the most appropriated choice was inevitably that of considering the percentage of maximal oxygen uptake. In addition, the exercise intensity range considered by the model is below (or up to) the anaerobic threshold, where the variability of lactate response to the same %VO_2max_ is substantially lower than at higher intensities, which further substantiate our choice.

The assumption to describe the changes in oxygen consumption (VO_2_) in terms of %VO_2max_ [20] from the beginning of the physical activity to the recovery period with a mono-exponential model, as in [26], was previously demonstrated to be valid in the case of exercise not-exceeding the LT. Conversely, when lactate starts to accumulate in the bloodstream, at least two kinetic components are required to characterize the dynamic response of %VO_2max_ [39, 40]. This improved description of changes in oxygen uptake (*PVO*_2max_) given by Eq (3) is used to model the dynamics of epinephrine concentration *C*_*E*_(*t*), allowing it to vary with *PVO*_2max_. For this purpose, we adapt and integrate a previously proposed model of epinephrine secretion and elimination [14] into the new computational model.

In the original KSC model the epinephrine secretion *C*_*E*_(*t*) is a function of WR. The parameters used to describe such dependence are fixed and, as a consequence, the model is not able to give an appropriate description of the dynamics of epinephrine in the case of different exercise intensities as well as not allowing to normalize the effort according to the individual exercise/functional capacity and anthropometric characteristics. Similarly to the original model, it is here postulated that changes in the hormone epinephrine affect plasma insulin concentration in the hormonal model as described by Eq (7). The epinephrine contribution on insulin changes is assumed to be bilinear (through *k*_5_ parameter) and is modeled similarly to the terms accounting for the insulin and the glucagon contributions.

The model parameter *k*_5_ is estimated by fitting experimental data of glucagon and insulin concentrations obtained from the study by Hirsch et al. [28] during a session of exercise. The low value for its *CV*% indicates the good level of precision of the estimate. The simulated dynamics of insulin and glucagon fit well the set of observations during the exercise period (gray zone in Fig 2). After the exercise period, both insulin and glucagon concentrations return to the same steady-state values *C*_*I*, 0_ and *C*_*G*, 0_ as in the KSC model. Moreover, as depicted in Fig 2, the residuals are centered on zero throughout the range of fitted values and are substantially normally distributed, thus guaranteeing the goodness of the estimate. Moreover, the sensitivity analysis shows that changes in insulin response are physiologically consistent to variations in the parameters T_v_, *k*_5_ and BW (see S1 Appendix in “Supporting information” for further details).

**Fig 13:**
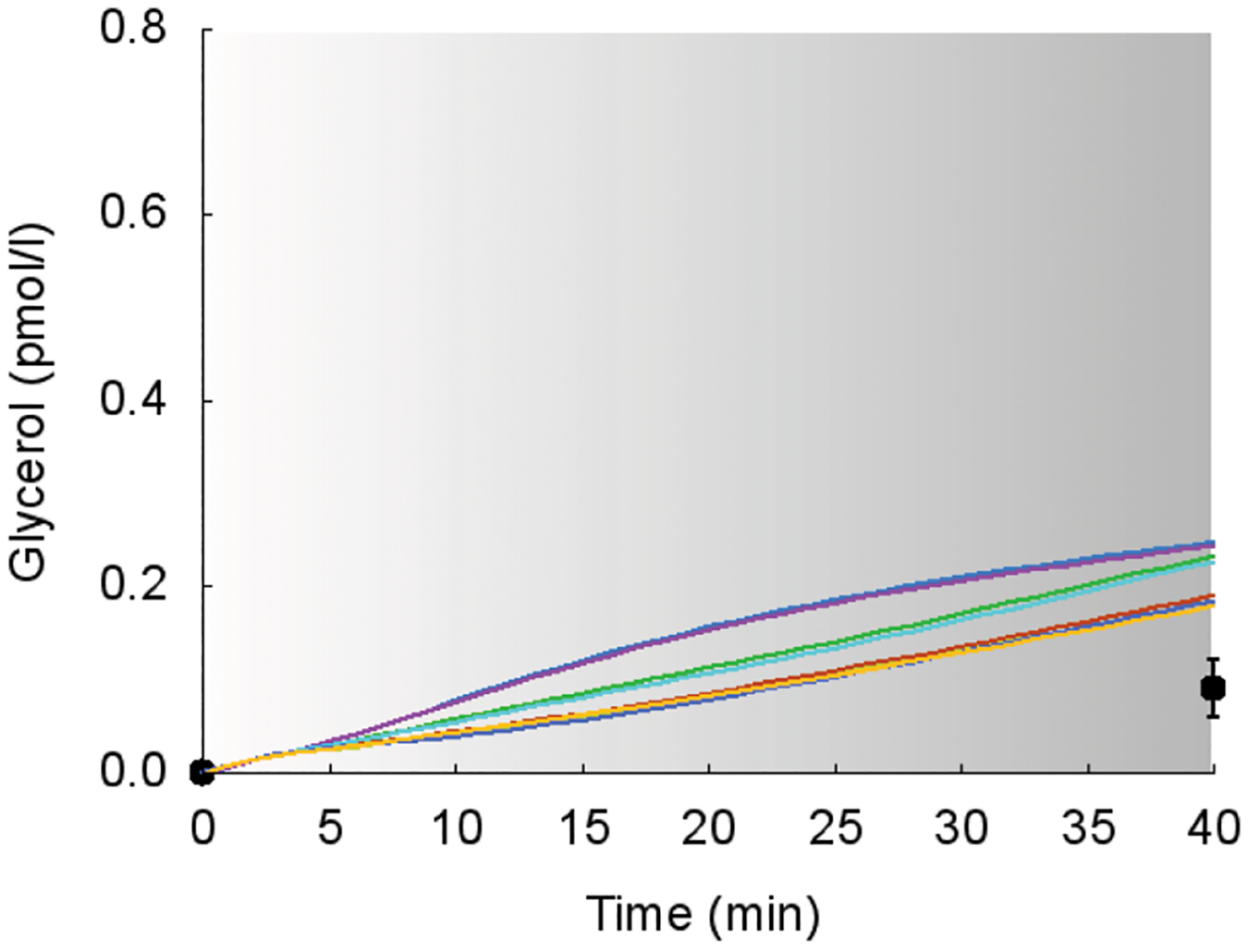
Dynamics of suprabasal plasma glycerol concentration in response to exercise. Model fit (colored lines) vs experimental data (circles) expressed as mean ± SE. The colored lines show the different simulated dynamic responses of the eight subjects from the study 2 [32]. The gray zone refers to the exercise period.

As stated before, our model is able to take into account the subjects’ variability as regards either the individuals’ characteristics or the exercise parameters. The experimental data sets used to validate our computational model are listed in Table 2. All the studies but one (namely study 2 by Wahren et al. [32] indicate the ranges of variability or express the data as mean ± standard deviation, standard error or standard error of the mean, thus providing maximum and minimum values, as reported in Table 2. As a consequence, we simulated the “artificial subjects’ responses by attributing them the characteristics corresponding to the combinations between these maximum and the minimum values. We used a red area to indicate that all the simulations of the individuals fall between these “extremal” cases, i.e., a range of responses. The study 2 by Wahren and colleagues is the only one that specifically indicates the characteristics of each of the eight subjects involved in the study 2. As an example, we reported in Fig 13 the simulations of glycerol concentration for each of such subjects (eight different curves) superimposed to experimental data from the study 2.

As evident from the figure, the simulations obtained for the eight subjects show similar dynamics, namely a monotonic growing trend. It is worth to be noted that in Fig 13 it is possible to observe the individual responses of the eight subjects from the study 2, while in Fig 7 panel C we reported the same dynamics using the red area visualization, thus meaning that individual responses data is present even if by using the red zone we show the range of the whole population responses.

### Physiological remarks

Modeling the effect of exercise on metabolic regulation is a challenging task as it is a complex phenomenon characterized by a myriad of interacting factors. The task is further complicated by the intra- and inter-individual variability of the acute and chronic responses to exercise. As a matter of fact, even after a careful standardization of the exercise stimulus, the same exercise session may lead to a variable response, and its repetition over time (i.e., training) to a wide range of adaptation levels. It is clear, that the biological variability linked to the hormonal and metabolic regulation during exercise is a factor that should be taken into account when judging mathematical models.

The data used to validate the model refers to subjects aged between 20 and 40 years, nevertheless the range of applicability is wider, as the metabolic response to moderate intensity exercise is similar, in relative terms, between individuals with different age. Moreover, published experimental data points regarding young individuals turned out to be inadequate to the purpose of validating the model. When the elderly population is concerned, it is difficult to discriminate the effect of aging *per se* from that of the pathologies associated with it, which makes it difficult to find studies describing the pure effect of the aging process. With regard to sex differences, we have reported the simulation results for only one study taking into consideration women. Experimental data on women is more difficult to obtain with respect to men, since women are subject to hormonal changes that affect their metabolic responses (they have to be generally tested in the early to midfollicular phase of the menstrual cycle). On the other hand, we have reported the simulations obtained from the study by Carter [34] describing the effects of exercise on men and women and we applied our model to the data from the study by Friedlander and colleagues [41] obtaining a good level of precision in simulating the concentrations of glycerol and FFA (data not shown), demonstrating that our computational model is able to capture the difference between men and women.

Considering this, the matching between the experimental data and those originated by the model is remarkable. We could say that deviations between experimental data and model simulations are physiologically consistent and quantitatively negligible.

The model well reproduces the response of blood glucose, and the related hormonal controlling factors, to various exercise conditions. Indeed, during moderate intensity endurance exercise the increased muscle glucose uptake is balanced by an augmented endogenous glucose production and glycemia is maintained, at least when the exercise is not very prolonged. In these conditions, the model correctly reproduces a decline in insulin level and the concomitant rise of glucagon, which together with epinephrine is an important regulator of the hepatic glucose production. With regards to the latter, the dominance of glycogenolysis over gluconeogenesis for hepatic glucose output during exercise, especially in the early exercise phase, is also nicely reproduced by the model. In this respect, the model also simulates opportunely the trend of the main gluconeogenic precursors lactate, glycerol and alanine. It correctly shows the rise of epinephrine and lactate during incremental exercise as well as their behavior at the start and during constant intensity submaximal exercise. On the other hand, the modeled glucagon dynamics during incremental exercise is not following the original data from Bloom et al., whereas it shows a good agreement with data from the study by Wahren. It should be considered, however, that this exercise modality is probably not the most appropriate for the comparison, as glucagon response is quite variable and depending on the specific exercise conditions, often showing small increases during short or moderate duration exercise or even a decrease at very high exercise intensities [42]. Differently, the lipolytic stimulus of moderate intensity exercise is also well depicted by the model, with the progressive increase of glycerol and FFA concentrations, which pairs with the reduction in insulin and the increase in epinephrine. Muscle glycogen is a classical parameter monitored to describe substrate metabolism during exercise at different intensities and durations. In this regard, the model is able to simulate the decline of muscle glycogen concentration during prolonged exercise, with a good accuracy with respect to the classical data from Gollnick et al [35].

Taken together, the accuracy of the simulated dynamics of the various variables considered substantiates the use of the proposed model as one of the few allowing the prediction of several aspects of the hormonal and metabolic responses to exercise.

### Future developments

Since it is widely recognized that physical activity is effective in preventing and/or treating major chronic metabolic diseases, a modeling tool able to take into account individual’s metabolic, anthropometric parameters and fitness status, can represent a valid support in the view of the development of diagnostic platforms for medical devices and patient-specific eHealth applications.

Further research objectives will include the implementation of other key aspects for a better description of the changes in the metabolism due to the individual’s lifestyle. Among such aspects, nutrition is clearly of paramount importance. We thus foresee the inclusion in the model of nutritional patterns, related caloric intake and nutrient absorption rates and modes. For this task, it will be necessary to add the nutritional regime, consisting of a model of nutrient intake, stomach emptying and absorption of macronutrients monomers in the gut, posing non trivial modeling and integrative challenges.

Another key model enhancement will be the the description and the integration of the changes in inflammatory markers of interest, assumed that inflammation is nowadays recognized as one of the major triggers of metabolic diseases [43, 44]. Such task could be carried out by integrating the model here described with an agent-based simulator of the immune system developed by some of us [45], finally realizing a multi-scale model possibly encompassing several biological levels of description, from the intracellular (i.e., gene regulation) level up to the cellular, organ and thus to the system-level dynamics.

In this view, the ability of the model to manage fundamental personal lifestyle parameters, combined with the availability of other computational models providing for nutrition, inflammation, immune system etc., can be the key to access integrated, multilevel, and patient-specific computational models used as diagnostic tools [46] aimed at improving the comprehension of both well-being and pathological state, including early shift to disease onset [47].

## Supporting information

S1 Appendix Sensitivity analysis. We have performed a sensitivity analysis to understand how the values of BW, T_v_ and *k*_5_ impact on the key variable plasma insulin concentration.
(TEX)

S2 Appendix The Kim computational model. Brief description of the whole-body computational model by Kim et al. [13].
(TEX)

S1 Fig Sensitivity analysis. Dynamics of insulin concentration for the sensitivity analysis in the first procedure.
(TIF)

S2 Fig Sensitivity analysis. Dynamics of insulin for the sensitivity analysis after estimating the parameter *k*_5_.
(TIF)

S3 Fig Sensitivity analysis. Dynamics of insulin for the sensitivity analysis in the second procedure.
(TIF)

S1 Table Metabolites and reactions. Metabolites and reactions in the computational model of fuel homeostasis by Kim et al. [13].
(TEX)
