# Supplementary figures and images for: Personalizing physical exercise in a computational model of fuel homeostasis
Source: PLoS Comput Biol. 2018 Apr 26;14(4):e1006073. doi: 10.1371/journal.pcbi.1006073 (PMC5919631; doi:10.1371/journal.pcbi.1006073)

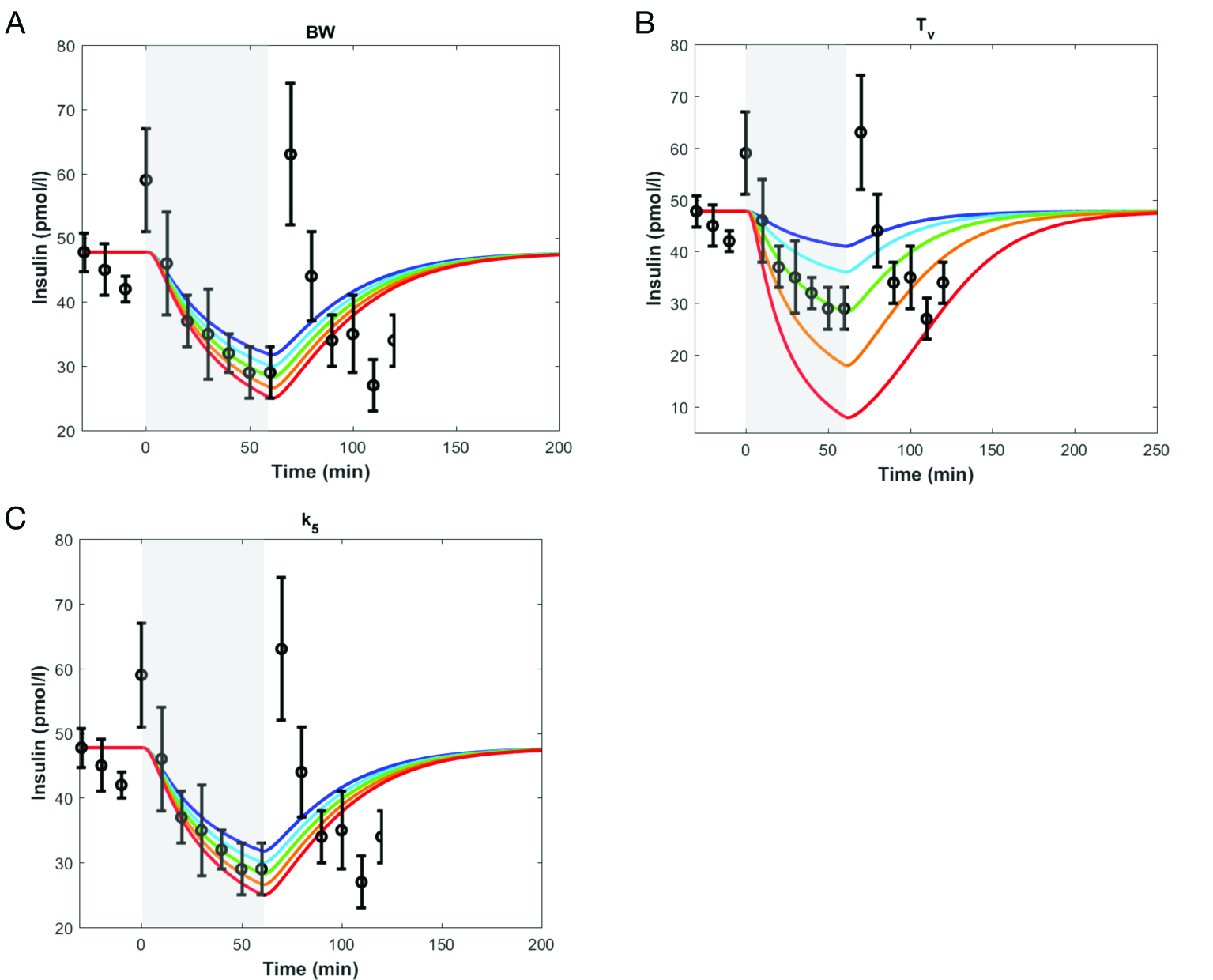

Supplement: S1 Fig — Dynamics of insulin concentration for the sensitivity analysis in the first procedure. (TIF) [file pcbi.1006073.s003.tif]

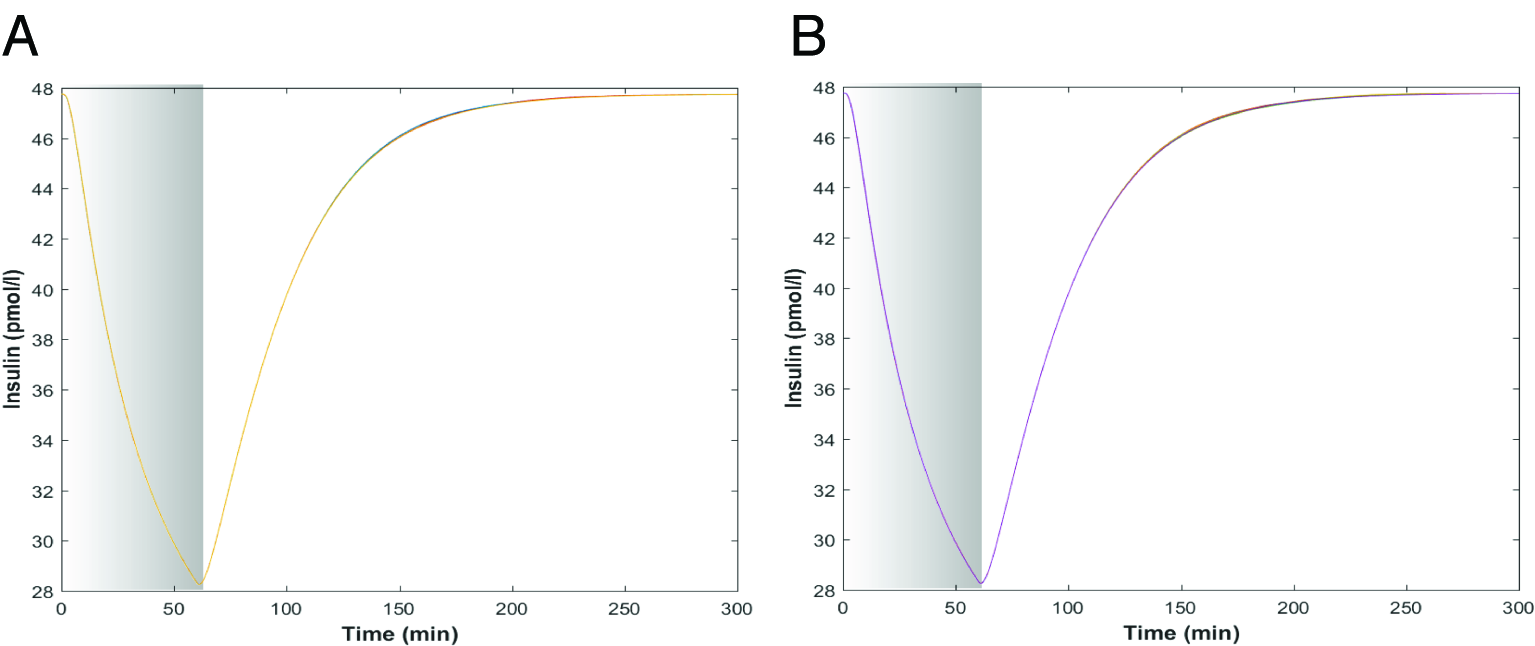

Supplement: S2 Fig — Dynamics of insulin for the sensitivity analysis after estimating the parameter k5. (TIF) [file pcbi.1006073.s004.tif]

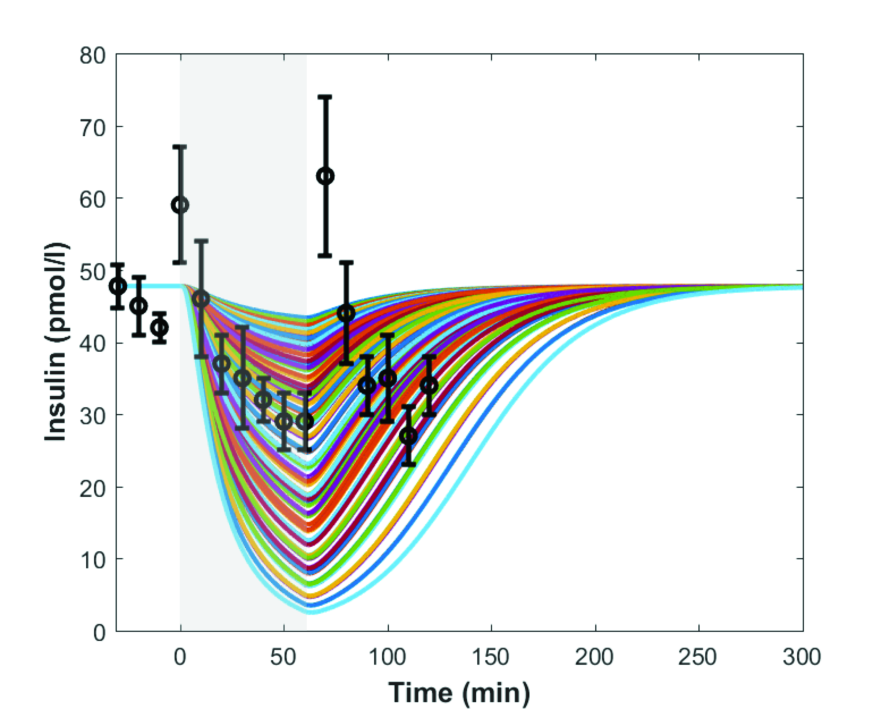

Supplement: S3 Fig — Dynamics of insulin for the sensitivity analysis in the second procedure. (TIF) [file pcbi.1006073.s005.tif]
